# Evaluation of an older adult medical school alumni mentorship program for medical students to learn about aging

**DOI:** 10.1093/geroni/igaf122.3581

**Published:** 2025-12-31

**Authors:** Micayla Flores, Emily Harris, Louise Aronson, Beth MacGillivray, Andrea Wershof Schwartz

**Affiliations:** Massachusetts General Hospital, Boston, Massachusetts, United States; Harvard Medical School, Harvard Medical School, Massachusetts, United States; Harvard Medical School, Boston, Massachusetts, United States; Harvard Medical School, Boston, Massachusetts, United States; Veteran’s Affairs, Jamaica Plain, Massachusetts, United States

## Abstract

Medical school training must prepare future clinicians to care for our aging population by providing them with skills and knowledge about older adults, including tools for avoiding the negative health impacts of ageism. AGE- PAIRS (Aging and Geriatrics Education: Physician Alumni Intergenerational Relationships with Medical Students) was conducted at Harvard Medical School from Aug 2024- June 2025, matching medical school students with older adult medical school alumni. The program consists of an introductory session, calls focused on 4 themes (medicine as an evolving profession, perceptions of aging, retirement, ageism), and a pre- reading or podcast. The program was evaluated using focus groups and a pre and post program survey that also assessed attitudes towards aging with a pre- validated questionnaire. 148 medical student- mentor dyads participated in the program. Before program initiation, there was no difference between students’ and mentors’ attitudes towards aging, and there was no change in the mentors’ attitudes towards aging following program completion. 78% mentors expressed the experience in the program was moderately, very, or extremely effective, and 81% mentors expressed the experience with their student was very or extremely positive. 92% mentors would like to mentor again. Next steps include post- program student evaluations, including a survey and focus groups. This highly- rated program proved to be a positive experience, providing a balance of career advising and insight into the aging experience, and offering a potentially powerful model for preparing students to care for older adults with empathy and excellence.

